# SFXN3 is Associated with Poor Clinical Outcomes and Sensitivity to the Hypomethylating Therapy in Non-M3 Acute Myeloid Leukemia Patients

**DOI:** 10.2174/1566523223666230724121515

**Published:** 2023-09-19

**Authors:** Yuxuan Dong, Fengbo Jin, Jing Wang, Qingsheng Li, Zhenqi Huang, Leiming Xia, Mingzhen Yang

**Affiliations:** 1 Department of Hematology, The First Affiliated Hospital of Anhui Medical University, Hefei, China;; 2 Anhui Public Health Clinical Center, Hefei, China

**Keywords:** Acute Myeloid Leukemia (AML), SFXN3 (Sideroflexin3), DNA methylation, epigenetics, hypomethylating therapy, outcome

## Abstract

**Background:**

DNA hypermethylation plays a critical role in the occurrence and progression of acute myeloid leukemia (AML). The mitochondrial serine transporter, SFXN3, is vital for one-carbon metabolism and DNA methylation. However, the impact of SFXN3 on the occurrence and progression of AML has not been reported yet.

**Objective:**

In this study, we hypothesized that SFXN3 indicates a poor prognosis and suggested tailored treatment for AML patients.

**Methods:**

We used GEPIA and TCGA repository data to analyze the expression of SFXN3 and its correlation with survival in AML patients. RT-qPCR was used to detect the SFXN3 level in our enrolled AML patients and volunteers. Additionally, Whole Genome Bisulfite Sequencing (WGBS) was used to detect the genomic methylation level in individuals.

**Results:**

Through the TCGA and GEPIA databases, we found that SFXN3 was enriched in AML patients, predicting shorter survival. Furthermore, we confirmed that SFXN3 was primarily over-expressed in AML patients, especially non-M3 patients, and that high SFXN3 in non-M3 AML patients was found to be associated with poor outcomes and frequent blast cells. Interestingly, non-M3 AML patients with high SFXN3 levels who received hypomethylating therapy showed a higher CR ratio. Finally, we found that SFXN3 could promote DNA methylation at transcription start sites (TSS) in non-M3 AML patients. These sites were found to be clustered in multiple vital cell functions and frequently accompanied by mutations in DNMT3A and NPM1.

**Conclusion:**

In conclusion, SXFN3 plays an important role in the progression and hypermethylation in non-M3 AML patients and could be a potential biomarker for indicating a high CR rate for hypomethylating therapy.

## 1 INTRODUCTION

Numerous studies have confirmed that DNA methylation is critical in the occurrence and progression of AML [1-4]. Therefore, hypomethylating therapy is wildly accepted in clinical practice and has achieved significant efficacy in AML treatment [5-7]. However, the key molecule that regulates tumor DNA methylation in AML patients is still largely unknown.

SFXN3 is an important mitochondrial serine transporter that is required for generating one-carbon units, which is critical in cellular metabolism, including one-carbon metabolism and DNA methylation in both cytosolic and mitochondrial branches [8, 9]. It has been reported that serine is converted into glycine in mitochondria through the mitochondrial serine transporter, which is necessary for the synthesis of S-adenosylmethionine (SAM) [10-14]. In the cytosolic branch, the synthesis of the methionine-SAM cycle occurs in a serine-derived ATP-dependent manner [15]. SAM is the primary methyl donor in DNA and RNA methylation [16, 17]. Thus, SFXN3 could promote one-carbon metabolism and the methionine-SAM cycle by activating serine transport and enhancing SAM synthesis. This indicates that SFXN3 plays an important role in regulating tumor genomic hypermethylation.

However, it has not been reported whether SFXN3 could affect the occurrence and progression of AML. We hypothesized that SFXN3 indicates a poor prognosis and suggested a tailored treatment for AML patients.

## 2 MATERIALS AND METHODS

### 2.1 Bio-informatics Database

Raw data of SFXN3 expression in AML patients were downloaded from The Cancer Genome Atlas (TCGA) database, which can be accessed at https://gdc-hub.s3.us-east-1.amazonaws.com/download/TCGA-LAML.htseq_fpkm.tsv.gz. Full metadata is also available. Gene Expression Profiling Interactive Analysis database (GEPIA) was used to obtain SFXN3 expression data for AML patients and compared controls (access the website at http://gepia.cancer-pku.cn/detail.php?gene = SFXN3). Integrated data from both GEPIA and TCGA databases were analyzed *via* the GEPIA analysis system and R software (R 4.2.1) to determine the overall survival of SFXN3 high and low AML patients.

### 2.2 Patients’ Characteristics and Bone Marrow Obtained from our Home Hospital

The detailed diagnostic and classified criteria for the involved AML patients were according to the 2016 WHO guideline, and the patients were stratified using the 2017 European Leukemia Network (ELN) prognostic classification. We included a total of 31 newly diagnosed AML patients (Supplementary Table **1**) treated in our center from January, 2022, to June, 2022. Additionally, we enrolled 23 healthy volunteers in the control group (Supplementary Table **2**). The bone marrow and necessary clinical information were collected and recorded. All patients understood and signed the informed consent forms approved by the Ethics Review Committee of the First Affiliated Hospital of Anhui Medical University.

### 2.3 The Expression of SFXN3 was Detected by RT-qPCR

Total RNA was extracted from the bone marrow of patients and volunteers using the RNAprep Pure Hi-Blood Kit from TIANGEN, and nucleic acid quantification equipment (Eppendorf, BioPhotometer D30, DEU) was used to detect the purity and concentration of RNA. Then, the total RNA was reverse transcribed into cDNA by a reverse transcription kit (Plus All-One 1^st^ Strand cDNA Synthesis Super Mix, Novoprotein), and qPCR (Agilent, Mx3000P, USA) was used to detect the SFXN3 expression. RNA primer was synthesized by Sangon Biotech (Supplementary Table **2**) and SYBR qPCR kit was purchased from Novoprotein (SYBR qPCR Super Mix Plus, Novoprotein). The relative expression of the gene is represented by 2^-ΔΔCT^.

### 2.4 Detection of Genomic Methylation Level by Whole Genome Bisulfite Sequencing (WGBS)

#### 2.4.1 WGBS Experimental Method

Genomic DNA was extracted from bone marrow using the TIANamp Blood DNA Kit (TIANGEN). DNA concentration and integrity were assessed by a NanoDrop 2000 spectrophotometer (Thermo Fisher Scientific, Waltham, MA, USA) and agarose gel electrophoresis. Then, the genomic DNA samples were added with a certain proportion of negative control (lambda DNA) and randomly interrupted at 200–300 bp. The interrupted DNA fragments were repaired at the end; an A-tail was added and ligated to the sequencing adaptor with all cytosines methylated. After bisulfite treatment by EZ DNA Methylation Gold Kits (Zymo Research, Irvine, CA, USA), the unmethylated C turned into U, while the methylated C remained unchanged. Finally, after PCR amplification and product purification, the final libraries were sequenced on the Illumina NovaSeq 6000 platform (Illumina Inc., San Diego, CA, USA), and 150 bp paired-end reads were generated. The sequencing and analysis were conducted by OE Biotech Co., Ltd. (Shanghai, China).

#### 2.4.2 WGBS Analysis Process

The raw reads were subjected to a quality check and then filtered by Fastp [18]. Clean reads were aligned to the human genome (GRCh38.p13) using Bismark [19]. The alignment result was next assessed by MethylKit [20] to determine the methylation level of each C site (from 0% to 100%). To ensure the accuracy of the results, the C sites with coverage of <10x were filtered out. The sequence feature near the methylated C was analyzed using the R package ggseqlogo [21]. Differentially methylated regions (DMRs) were subsequently analyzed using MethylKit, and the parameter was set as “myobj, win.size = 1000, step.size = 1000.” The significant DMRs were identified by the threshold of a *p*-value ≤ 0.05 as well as the absolute delta cutoff between the two groups ≥ 10%. The promoter region was defined as 4000 bp upstream of the TSS of a gene. The overall methylation level of the promoter region for every gene was calculated, and the *P*-value between the two groups was calculated using the t-test. GO and KEGG pathway enrichment analyses of DMRs related genes were performed using R based on the hypergeometric distribution.

### 2.5 Statistical Methods

Quantitative variables were presented in mean±SD and analyzed using SPSS 26.0. The means between the two groups, which corresponded to normal distribution and homogeneous variance were compared using the t-test (T-test), otherwise, using the Mann-Whitney U test (Mann-Whitney U test). Analysis of variance (ANOVA) was used to compare the means among three or more groups corresponding to the normal distribution and homogeneous variance, otherwise, using the Kruskal-Wallis test (K-W test). Pearson Chi-square test and Fisher’s exact test were used to compare the proportions between two or more independent groups. *p* < 0.05 means the difference is statistically significant.

## 3 RESULTS

### 3.1 SFXN3 is Significantly Highly Expressed in Non-M3 AML Patients and is Associated with Poor Clinical Outcomes

**Fig. (1):**
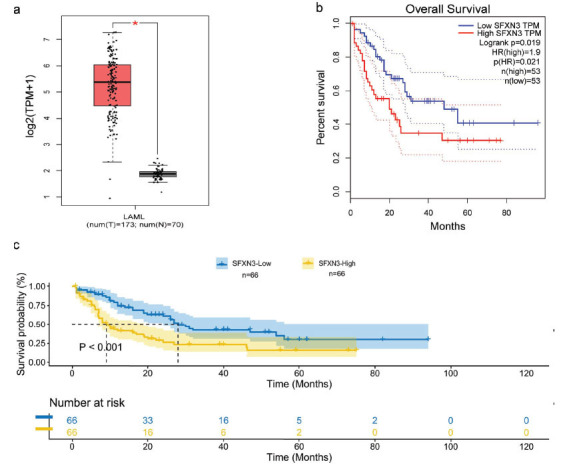
SFXN3 is enriched in AML patients and indicates a poor OS. (**a**) The expression of SFXN3 was significantly higher in AML patients (n = 173) than that in healthy individuals (n = 70) (*p* < 0.01). (Data was obtained from the GEPIA database). (**b** and **c**) AML patients with high SFXN3 expression had significantly short overall survival. (Data was obtained and analyzed from GEPIA (*p* = 0.019) and TCGA databases (*p* < 0.01), respectively).

To evaluate whether SFXN3 expression is related to the occurrence and progression of AML, we investigated the relationship through online databases. We found that the expression of SFXN3 was significantly higher in 173 AML patients than that in 70 healthy individuals (*p* < 0.01) (Fig. **1a**), according to data from GEPIA. Furthermore, we studied the overall survival (OS) of AML patients with high and low SFXN3 expressions, separated according to the median SFXN3 expression in the GEPIA and TCGA databases, respectively. Data from 106 AML cases in GEPIA (*p* = 0.019) and 132 cases in TCGA (*p* < 0.01) showed that AML patients with high SFXN3 expression had significantly shorter OS than those of patients with low SFXN3 (Figs. **1b** and **c**). The results indicated that high SFXN3 expression was significantly detected in AML patients, and that high SXFN3 expression in AML patients was associated with poor OS.

**Fig. (2):**
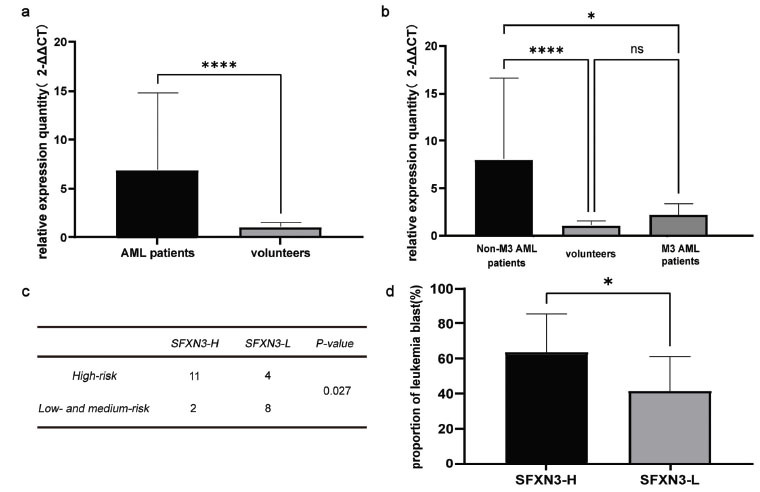
SFXN3 is preferentially over-expressed in non-M3 AML patients and is associated with high-risk and frequent blasts. (**a**) SFXN3 was over-expressed in AML patients compared with volunteers (*p* < 0.05). (**b**) SFXN3 was over-expressed in non-M3 AML patients compared with both M3 AML patients (*p* < 0.05) and volunteers (*p* < 0.01); however, no significant differences were found between M3 patients and volunteers (*p* > 0.05). (**c**) High SFXN3 non-M3 AML patients suffered a poor prognosis of high-risk (*p* < 0.05). (**d**) The proportion of leukemia blasts in the SFXN3-H group was significantly higher than that in the SFXN3-L group (*p < 0.05 ****p< 0.01).

Next, we collected bone marrow from 31 newly diagnosed AML patients and 23 volunteers from our home hospital and detected the expression of SFXN3. Our findings confirmed that SFXN3 was significantly higher in AML patients (6.89 ± 7.74) than in volunteers (1.08 ± 0.43) (*p* < 0.01) (Fig. **2a**). Furthermore, we investigated the variant SFXN3 expression in M3 and non-M3 AML patients and found that SFXN3 was preferentially overexpressed in non-M3 AML patients (8.02 ± 8.20) than in M3 patients (2.17 ± 1.12) (*p* < 0.05) and volunteers (1.08 ± 0.43) (*p* < 0.01). However, no significant changes in SFXN3 expression were demonstrated in M3 AML patients (2.17 ± 1.12) and volunteers (1.08 ± 0.43) (*p* > 0.05) (Fig. **2b**). These data consistently illustrated that SFXN3 was over-expressed in AML patients, especially in non-M3 AML patients.

According to the median SFXN3 expression, 25 newly diagnosed non-M3 AML patients were divided into high SFXN3 expression (SFXN3-H) group (n = 13) and low SFXN3 expression (SFXN3-L) groups (n = 12). We found that the ratio of high-risk patients in the SFXN3-H group was significantly higher than that of the SFXN3-L group (*p* < 0.05) (Fig. **2c**). The percentage of blast cells in the SFXN3-H group (63.42% ± 21.50%) was significantly higher than that of the SFXN3-L group (41.54% ± 17.88%) (*p* < 0.05) (Fig. **2d**). These findings suggested that the high SFNX3 expression indicates worse risk and tumor burden, further confirming the poor clinical prognosis of non-M3 AML patients.

### 3.2 High SFXN3 Expressed Non-M3 AML Patients Potentially Benefit from Hypomethylating Therapy

We further explored the relationship between SFXN3 expression and the efficacy of hypomethylating therapy. In the SFXN3-H group, all 6 patients who received hypomethylating therapy achieved complete remission (CR), while only 2 out of 7 patients who did not receive hypomethylating therapy got CR. A significantly increased CR rate was observed in non-M3 AML patients with high SFXN3 expression who received hypomethylating therapy, compared to those without hypomethylating treatment (*p* < 0.05). In the SFXN3-L group, no statistical difference was found in the CR rate with or without hypomethylating therapy (*p* > 0.05). Thus, high SFXN3 expression in non-M3 AML patients may indicate potential benefit from hypomethylating therapy (Table **1**).

### 3.3 SFXN3 Promotes DNA Methylation in Non-M3 AML Patients at Transcription Start Sites (TSS), which Clustered in Multiple Vital Cell Functions and Accompanied by Mutations in DNMT3A and NPM1

**Fig. (3):**
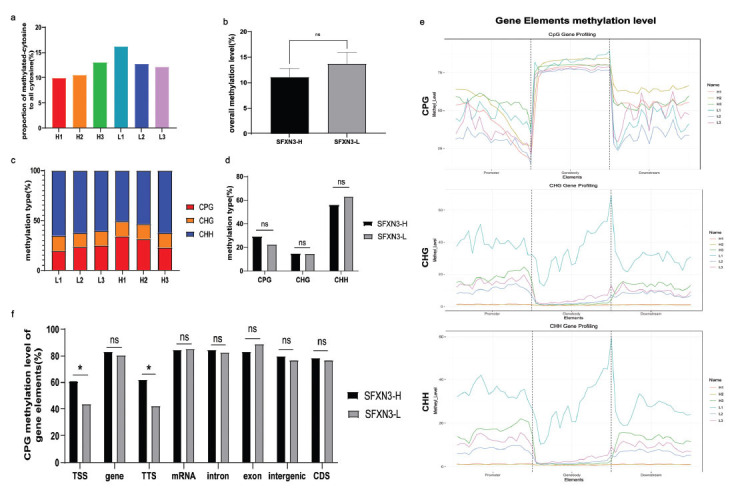
SFXN3 can promote the CpG methylation level at the transcription start sites (TSS) in non-M3 AML patients. (**a**), (**b**) There was no difference in methylated cytosine ratio between the SFXN3-H and SFXN3-L groups (*p* > 0.05). (**c**) The proportion of three methylation types (CpG, CHG, and CHH) in each sample. (**d**) The CpG methylation ratio in the SFXN3-H group was higher than that in the SFXN3-L group, while the CHG and CHH methylation ratio was found with no significant difference in the two groups (*p* > 0.05). (**e**) The distribution of three methylation types (CpG, CHG, and CHH) in gene elements of SFXN3-H and SFXN3-L groups. (**f**) The methylation ratio of gene elements in the SFXN3-H and SFXN3-L groups. The methylation level at transcription start sites (TSS) in the SFXN3-H group was significantly higher than that in the SFXN3-L group (*p* < 0.05). In addition, the methylation ratio of the SFXN3-H group also increased at transcription termination sites (TTS) (*p* < 0.05). However, there was no significant difference between the two groups in other gene regions (*p< 0.05).

To our knowledge, SFXN3 is critical in generating one-carbon units and DNA methylation in both cytosolic and mitochondrial branches. Our data indicated that non-M3 AML patients with high SFXN3 expression could benefit from hypomethylating therapy. We hypothesized that SFXN3 might be involved in non-M3 AML progression *via* promoting aberrant DNA methylation. Thus, we conducted genomic methylation sequencing by whole genome bisulfite sequencing (WGBS) in high and low SFXN3-expressed non-M3 AML patients. We found no difference in methylated cytosine ratio between the SFXN3-H (11.13% ± 1.34%) and SFXN3-L (13.70% ± 1.79%) groups (Figs. **3a** and **b**). Additionally, the ratio of CpG, CHG, and CHH showed no difference in the SFXN3-H (29.40% ± 4.83%; 14.74%± 0.23%; 55.86% ± 5.05%) and SFXN3-L (22.55% ± 4.83%; 14.73% ± 0.48%; 62.72% ± 2.02%) groups (Figs. **3c** and **d**). However, we observed that the CpG methylation ratio was considerably higher in the SFXN3-H group than that in the SFXN3-L group (*p* < 0.05), whereas no significant increase was monitored in CHG and CHH (Fig. **3e**). Furthermore, the sequencing data revealed that CpG methylation at transcription start sites (TSS) was up-regulated in the SFXN3-H group (60.87% ± 4.39%) than in the SFXN3-L group (43.84% ± 7.03%) (Fig. **3f**). TSS is crucial in controlling gene transcription and expression, and aberrant methylation plays a significant pathophysiological role in AML [22-24]. Our findings suggest that SFXN3 might promote a poor prognosis and outcome by inducing aberrant methylation of TSS.

**Fig. (4):**
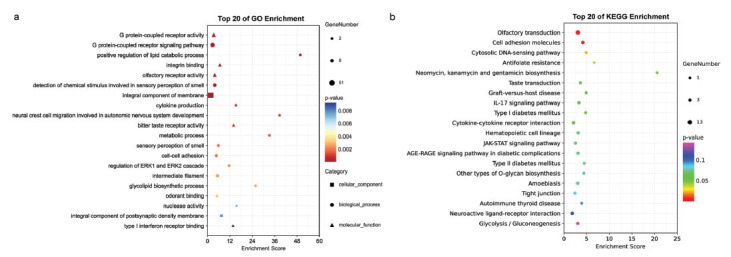
(**a**, **b**) Multiple vital cell functions are highlighted by the enrichment of TSS hypermethylation, including tumor cellular signal transduction, immunological recognition, tumor micro-environment, metabolism of nutriment, *etc*.

We clustered 163 genes that showed a significant increase in methylation in gene promoters of over 10% in GO and KEGG enrichment analysis. The enrichment of TSS hypermethylation highlighted multiple vital cell functions, including tumor cellular signal transduction, immunological recognition, tumor micro-environment, metabolism of nutriment, *etc*. (Fig. **4**).

**Fig. (5):**
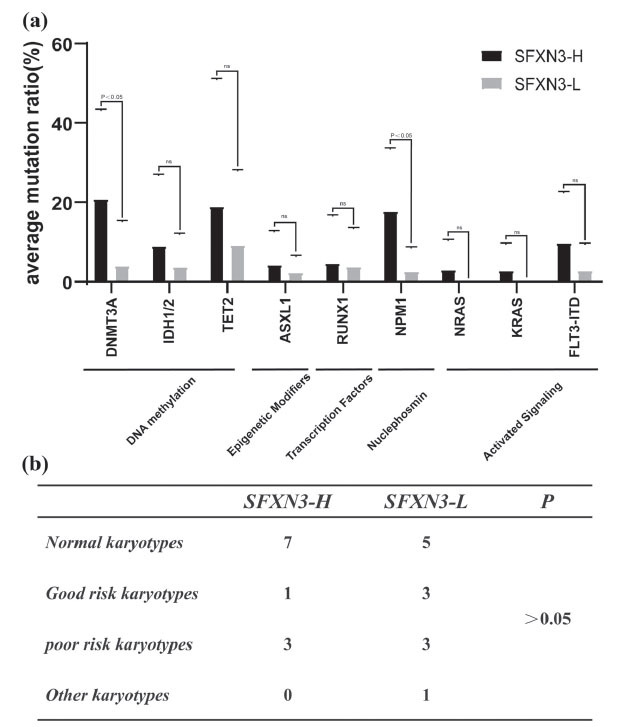
High SFXN3 is accompanied by mutations in DNMT3A and NPM1 in non-M3 AML patients. (**a**) By comparing the gene mutation sequencing of the SFXN3-H and SFXN3-L groups, we discovered that gene mutations with poor prognosis were frequently found in SFXN3-H group, and DNMT3A and NPM1 were statistically significant (*p* < 0.05). (**b**) There was no significant variation in the chromosomal karyotype between the SFXN3-H and SFXN3-L groups.

Finally, we explored the relationship of SFXN3 with the molecular and cytogenetic characteristics of non-M3 AML patients. By comparing the gene mutation sequencing of the SFXN3-H and SFXN3-L groups, we discovered that frequently mutated genes with poor prognosis were found in the SFXN3-H group. Specifically, DNMT3A and NPM1 were statistically significant (20.65% ± 22.64% *vs* 3.5% ± 11.61%; 17.15% ± 16.68% *vs* 2.03% ± 6.74%) (*p* < 0.05) (Fig. **5a**). There was no significant variation in the chromosomal karyotype between the SFXN3-H and SFXN3-L groups (Fig. **5b**).

In conclusion, SFXN3 promoted DNA methylation at transcription start sites (TSS) in non-M3 AML patients. These TSS were clustered in multiple vital cell functions and were accompanied by mutations in DNMT3A and NPM1.

## 4 DISCUSSION

Recent studies have shown that gene mutations and disorders play a key role in diagnosing and prognosis of new AML patients [25, 26]. Mono- and combination therapies with the backbone of hypomethylating agents have demonstrated satisfactory efficacy in clinics [5, 7, 27]. Mutations in methylation-related genes, such as TET-2 and IDH1/2, can enhance genomic methylation levels and are associated with poor disease prognosis [25, 28-30]. However, the trigger and pathophysiological processes of DNA methylation are unclear. Our study reported that SFXN3 was over-expressed in non-M3 AML patients and was associated with poor OS, high risk, and increased blast. Next, we observed frequent DNA methylation at TSS and TTS in high SFXN3 AML patients, usually accompanied by DNMT3A and NPM1 mutation. Our data suggested that SFXN3 may be a novel potential biomarker for the non-M3 AML patient prognosis. Previous research has already shown that SFXN3 promotes one-carbon metabolism and determines the prognosis of many other tumors [8, 31, 32]. Our findings confirmed that SFXN3 promotes tumor hypermethylation in multiple cancers.

Numerous studies have established that DNA hypermethylation, especially occurring at TSS, plays a substantial role in AML [22-24] and always indicates poor survival of AML patients [1, 2, 4, 33]. Although hypomethylating treatments have achieved satisfactory efficacy in the clinics, the efficacy has been observed to have significant heterogeneity, with only about 30%-40% of the patients responding to hypomethylating treatment and the OS being significantly reduced in high-risk patients [7, 34, 35]. Thus, key biomarkers are needed to be explored to guide personalized hypomethylating strategies [35, 36]. We detected significantly frequent DNA methylation at TSS and TTS in high SFXN3 non-M3 AML patients and identified that these patients could benefit from hypomethylating therapy. Our data indicated that non-M3 AML patients with high SFXN3 could benefit from hypomethylating therapy due to TSS methylation, which provides a novel and important biomarker for optimizing the option of a hypomethylating strategy. EZH2, DNMT3A, IDH1/2, and TET2 have been associated with DNA methylation and hypomethylating therapy. Among these genes, EZH2 and DNMT3A are related to the activity of DNMT *in vivo*, while TET2 and IDH1/2 take part in the process of DNA demethylation. Mutations in these genes show a higher remission rate to hypomethylating therapy in AML patients [25, 37-39]. Different from previously reported genes, SFXN3 mainly promotes DNA methylation by activating serine transport and determines whether patients can benefit from hypomethylating therapy, providing a new theoretical basis for hypomethylating therapy.

SFXN3 is a novel and seldom evaluated serine transporter in cancer cell development [9]. Previous studies revealed its effect on mitochondrial serine transport and one-carbon metabolism [8], which indicated that SFXN3 might be regulating hypermethylation in various tumor genomes. However, whether the hypermethylation of AML is partially generated by SFXN3 has not been reported yet. Through our study, we demonstrated that high SXFN3 non-M3 AML patients were detected with frequent DNA methylation, especially enriched in TSS. The WGBS analysis indicated that the TSS genes were clustered in multiple vital cell functions. Genomic hypermethylation was involved in tumor cellular signal transduction, immunological recognition, tumor micro-environment, metabolism of nutriment, *etc*. These findings indicated that the regulation of SFXN3 in tumors is multifaceted. DNA hypermethylation occurs in multiple genes, some of which are associated with G-protein-coupled receptors, appear in many cancers, and affect the efficiency of chemotherapy drugs [40, 41]. Some tumor suppressor genes are affected by hypermethylation and promote tumor genesis and invasion through the ERK signaling pathway [42]. Additionally, DNA hypermethylation can control cellular immune responses [43, 44], change IL-17A genetic polymorphisms [45], result in extracellular instability [46], influence glycose metabolism and cell proliferation [47], and participate in the development of various cancers.

Interestingly, we found that the high SFXN3 was accompanied by mutations in DNMT3A and NPM1. AML patients harboring DNMT3A mutations were found with poor OS and prognosis and satisfied response to hypomethylating therapy [39, 48]. Nevertheless, the global DNA methylation status in the DNMT3A mutant genotype in AML has not been fully clarified and the evidence is controversial [25]. Research revealed that DNMT3A encodes DNA methyltransferases that catalyze the addition of a methyl group to the cytosine residue of CpG dinucleotide [37], and ﻿DNMT3A mutation could present in the hematopoietic stem/progenitor cells of primary blasts and mature cells after remission [49-51].

However, the relationship between SFXN3-induced methylation enrichment and DNMT3A mutation needs further exploration. Nucleophosphorin1 (NPM1) is a chaperone protein that functions as a tumor suppressor gene in a number of cellular processes [51]. Its mutation frequently coexists with DNMT3A and FLT3-ITD mutations [28] and affects the activity of TP53 regulatory protein [52]. The association of SFXN3 with these gene mutations further suggests its impact on the prognosis of non-M3 AML patients and their sensitivity to hypomethylating therapy.

## CONCLUSION

Our study showed that the mitochondrial serine transporter SFXN3 is significantly highly expressed in non-M3 AML patients and is associated with poor clinical outcomes. The expression of SFXN3 could indicate whether the patients can benefit from the hypomethylating therapy or not. Furthermore, we confirmed that SFXN3 promotes DNA methylation in non-M3 AML patients at transcription start sites (TSS), which clustered in multiple vital cell functions and accompanied by mutations in DNMT3A and NPM1, indicating a potential biomarker for evaluating tumor methylation enrichment and predicting the prognosis of the disease.

## Figures and Tables

**Table 1:** High SFXN3 expressed non-M3 AML patients potentially benefit from hypomethylating therapy.

-	**With Hypomethylating Therapy**		**Without Hypomethylating Therapy**		***p*-value**
	**CR**	**Non-CR**	**CR**	**Non-CR**	
**SFXN3-H**	**6**	**0**	**2**	**5**	**0.039**
**SFXN3-L**	**5**	**1**	**4**	**1**	**0.887**

**Note:** In the SFXN3-H group, 6/6 patients who were administrated hypomethylating therapy achieved complete remission (CR), but 2/7 patients got CR when treated without hypomethylating therapy. A significantly higher CR rate was observed in non-M3 AML patients with high SFXN3 expression who were treated with hypomethylating therapy than in treatment without hypomethylating therapy (*p* < 0.05). In the SFXN3-L group, no statistical difference was found in the CR rate with or without hypomethylating therapy (*p* > 0.05).

## Data Availability

The data supporting the findings of the article is available in the GEPIA and the TCGA repository at http://gepia.cancer-pku.cn/detail.php?gene=SFXN3 and https://gdc-hub.s3.us-east-1.amazonaws.com/download/TCGA-LAML. htseq_fpkm.tsv.gz; Full metadata and https://gdc-hub.s3.us-east-1.amazonaws.com/download/TCGA-LAML.survival.tsv; Full metadata

## LIST OF ABBREVIATIONS

AML: Acute Myeloid Leukemia
ANOVA: Analysis of Variance
CR: Complete Remission
DMRs: Differentially Methylated Regions
ELN: European Leukemia Network
GEPIA: Gene Expression Profiling Interactive Analysis Database
NPM1: Nucleophosphorin1
OS: Overall Survival
SAM: S-adenosylmethionine
TCGA: The Cancer Genome Atlas
TSS: Transcription Start Sites
TTS: Transcription Termination Sites
WGBS: Whole Genome Bisulfite Sequencing

## STANDARDS OF REPORTING

STROBE guidelines were followed.
